# Discriminability of Single and Multichannel Intracortical Microstimulation within Somatosensory Cortex

**DOI:** 10.3389/fbioe.2016.00091

**Published:** 2016-12-02

**Authors:** Cynthia K. Overstreet, Randall B. Hellman, Ruben D. Ponce Wong, Veronica J. Santos, Stephen I. Helms Tillery

**Affiliations:** ^1^SensoriMotor Research Group, School of Biological and Health Systems Engineering, Arizona State University, Tempe, AZ, USA; ^2^Biomechatronics Lab, Department of Mechanical and Aerospace Engineering, Arizona State University, Tempe, AZ, USA

**Keywords:** intracortical microstimulation, tactile, proprioception, sensation, prosthesis

## Abstract

The addition of tactile and proprioceptive feedback to neuroprosthetic limbs is expected to significantly improve the control of these devices. Intracortical microstimulation (ICMS) of somatosensory cortex is a promising method of delivering this sensory feedback. To date, the main focus of somatosensory ICMS studies has been to deliver discriminable signals, corresponding to varying intensity, to a single location in cortex. However, multiple independent and simultaneous streams of sensory information will need to be encoded by ICMS to provide functionally relevant feedback for a neuroprosthetic limb (e.g., encoding contact events and pressure on multiple digits). In this study, we evaluated the ability of an awake, behaving non-human primate (*Macaca mulatta*) to discriminate ICMS stimuli delivered on multiple electrodes spaced within somatosensory cortex. We delivered serial stimulation on single electrodes to evaluate the discriminability of sensations corresponding to ICMS of distinct cortical locations. Additionally, we delivered trains of multichannel stimulation, derived from a tactile sensor, synchronously across multiple electrodes. Our results indicate that discrimination of multiple ICMS stimuli is a challenging task, but that discriminable sensory percepts can be elicited by both single and multichannel ICMS on electrodes spaced within somatosensory cortex.

## Introduction

Dexterous functional use of the hand depends upon tactile and proprioceptive feedback that relays information from the periphery to the central nervous system. The accuracy, precision, and speed of finger movements suffer greatly when these feedback mechanisms are absent (Sollerman and Ejeskär, 1995; Rosén, 1996). In current motor neuroprosthetic systems, the complete absence of sensory feedback from the limb forces prosthesis users to rely on visual feedback to monitor the position of the prosthesis and its interactions with the environment. The addition of tactile and proprioceptive feedback to these devices is expected to significantly improve the control of motor neuroprostheses (Shannon, 1976; Childress, 1980; Dhillon and Horch, 2005; Schwartz et al., 2006; Walker et al., 2015).

The major challenge in incorporating sensory feedback into neuroprostheses is delivering robust, easily interpreted artificial sensations to the prosthesis user. Significant recent advances have shown that interfaces with peripheral nerves can provide a robust and stable sensory percept that is useful for movement (Ortiz-Catalan et al., 2014; Raspopovic et al., 2014; Tan et al., 2014; Oddo et al., 2016). However, in many cases, these interfaces are not possible. For example, in spinal cord injury and peripheral neuropathies, functional peripheral nerves may not exist, thus creating a need for direct stimulation of central structures like somatosensory cortex. Intracortical microstimulation (ICMS) is one promising method for providing this input. Humans who experience electrical stimulation in this region of the brain report a variety of tactile and proprioceptive sensations (Libet et al., 1964; Libet, 1973). The body location of the sensation elicited by ICMS corresponds to the region of the sensory homunculus containing the stimulating electrode. The strength or intensity of the sensory percepts induced by ICMS can be modified by changing the amplitude, frequency, or timing between consecutive pulses of electrical stimulation (Romo et al., 1998; O’Doherty et al., 2011; Berg et al., 2013; Kim et al., 2015).

To encode functionally relevant sensations, a neuroprosthetic limb must be capable of transducing several independent streams of sensory information related to multiple locations on the prosthesis. To date, the main focus of somatosensory ICMS studies has been to deliver discriminable signals to a single location in cortex that vary in timing or intensity. ICMS within visual and auditory cortex has demonstrated that multiple discrete sensations can be evoked by stimulation of adequately spaced single electrodes (Maldonado and Gerstein, 1996; Schmidt et al., 1996; Otto et al., 2005; Deliano et al., 2009). However, the repetition, order, and timing of stimulation on nearby electrodes can modify the sensations perceived in response to stimulation on a single electrode (Penfield and Boldrey, 1937; Penfield and Welch, 1949; Bak et al., 1990; Schmidt et al., 1996; Tehovnik et al., 2005). Additionally, the percepts evoked by consecutive stimulation on single electrodes and simultaneous stimulation across multiple electrodes may differ substantially (Schmidt et al., 1996). These factors suggest that expanding the number of independent streams of artificial tactile and proprioceptive feedback *via* ICMS may not be a trivial task.

In this study, we investigated the discriminability of single and multichannel ICMS within the somatosensory cortex of a non-human primate. As the animal performed a change-detection task, we delivered single-channel stimulation on multiple electrodes spaced across somatosensory cortex. Additionally, we delivered patterns of multichannel ICMS, derived from a tactile sensor, simultaneously across three electrodes in somatosensory cortex. These methods allowed us to probe the ability of ICMS to evoke multiple unique and functionally relevant sensations.

Our results indicate that discriminating between multiple ICMS stimuli within somatosensory cortex is a challenging task. Accuracy was greatest for the two-channel single-electrode stimulation case, but even after the subject was well trained, performance did not exceed chance levels during some experimental sessions. Increasing the number of independent stimulation electrodes to three further increased the error. During patterned multichannel stimulation across three electrodes, only matching stimuli were consistently and accurately identified by the animal. In this work, we have demonstrated that both single and multichannel ICMS delivered to somatosensory cortex can produce discriminable sensations. We discuss some of the factors that likely contribute to discriminability of tactile sensations and their implications for delivering functionally relevant sensations for neuroprosthetic devices *via* ICMS.

## Materials and Methods

### Recording Chamber and Electrode Placement

All protocols were approved and monitored by the Arizona State University Institutional Animal Care and Use Committee and conformed to the standards within the “Guide for the Care and Use of Laboratory Animals” (Committee for the Update of the Guide for the Care and Use of Laboratory Animals and National Research Council, 2011).

During an aseptic surgical procedure, a custom recording chamber (McAndrew et al., 2012) was affixed to the skull of a non-human primate (*Macaca mulatta)* over a craniotomy exposing the central sulcus. During daily experimental sessions, the cap of the chamber was removed, and a microelectrode drive (NaN Instruments) was used to advance several tungsten microelectrodes (200 μm diameter, FHC) into the cortex. This drive includes a grid, which limits electrodes to positions on an *X*–*Y* grid separated by 1 mm in each direction. Neural recordings (RA16PA preamplifier and RZ5 processor, Tucker-Davis Technologies) were used to monitor the position of the electrode tip as it entered cortical tissue.

**Figure 1:**
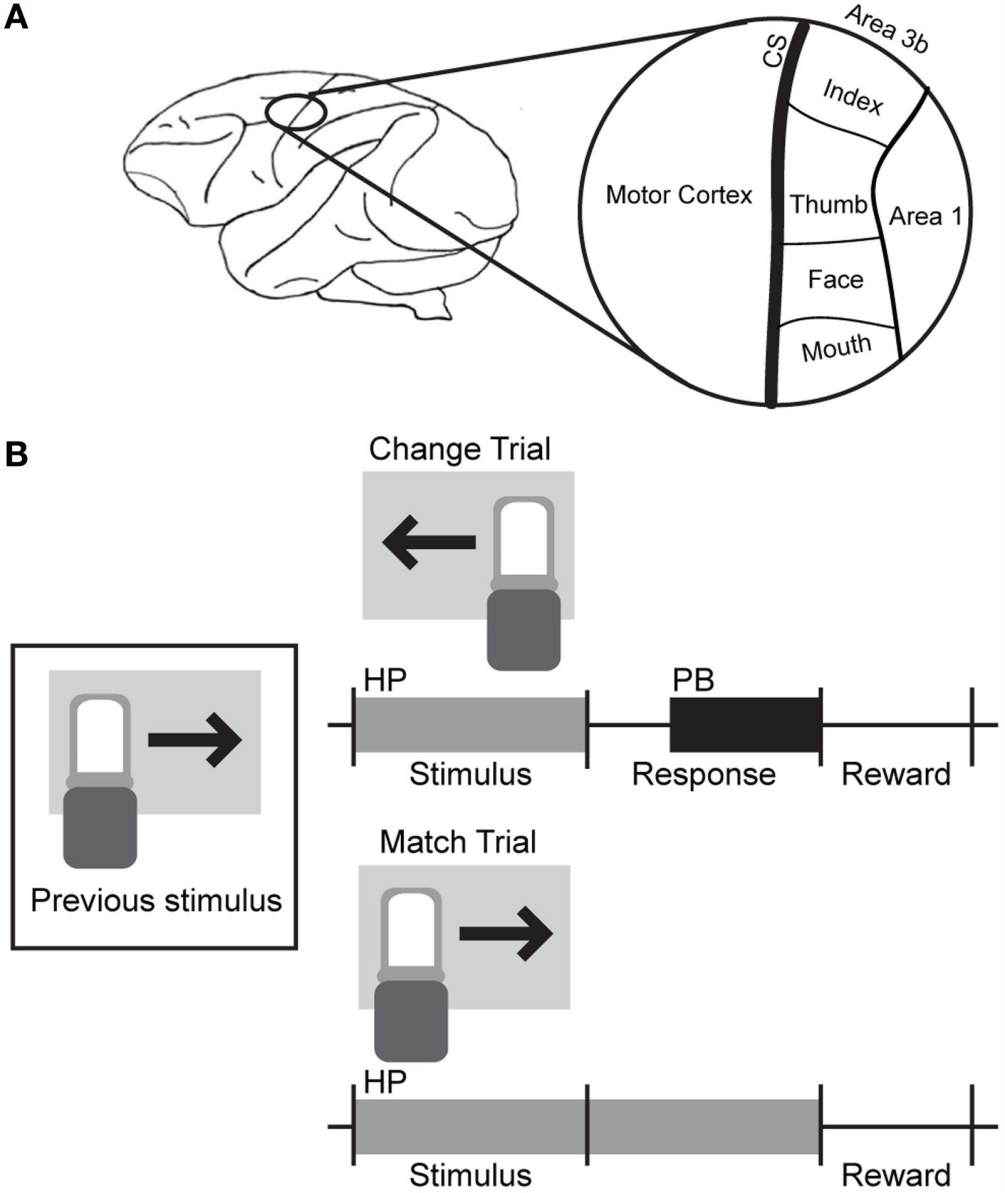
**Basic electrode and behavioral task layout**. **(A)** The craniotomy and recording chamber were placed over the left central sulcus. Electrodes placed within regions marked as Area 3b exhibited small receptive fields with defined edges, while larger receptive fields with gradual borders were observed in the region marked Area 1. **(B)** A change-detection task was used to probe the discriminability of ICMS of somatosensory cortex. Blocks of different types of stimuli (pictures, sounds, single-channel ICMS, or multichannel ICMS) were presented. During multichannel ICMS trials, a 1-s train of multichannel stimulation, corresponding to the movement of a sensor in one of four or eight directions, was delivered across three microelectrodes after a holdpad (HP) was depressed. If the stimulus was different from that delivered during the previous trial, the animal was required to release the holdpad and press a button (PB); if the stimulus was the same as the previous trial, the holdpad had to remain continually depressed until the end of the trial. A juice reward was delivered for some subset of correct trials (50–100% probability of reward) during the intertrial interval.

Most electrodes were driven to a depth of 0.3–1.0 mm beyond initial contact with the cortex. Receptive fields were identified for each electrode location. The chamber contained the central sulcus, some superficial regions with small receptive fields putatively identified as Area 3b, and a region with larger, overlapping receptive fields that likely corresponds to somatosensory cortical areas 1 and 2. Motor activity was also observed on some electrodes near the rostral edge of the chamber. An overview of the chamber location and estimates of the organization of the underlying cortical tissue are shown in Figure 1A.

### Behavioral Task

The non-human primate performed a simple change-detection task to report the discriminability of sensations elicited by ICMS in somatosensory cortex. The basic structure of this task is outlined in Figure 1B.

The animal was seated in a primate chair in front of a computer monitor and initiated trials by placing its hand on a holdpad (HP). A visual stimulus (2.5″ × 4″ color picture of a single object) was presented, and the animal was trained to press a button (PB) if the stimulus was different from that presented in the previous trial. The animal’s hand was required to remain on the HP until completion of the trial if the stimuli were the same. Releasing the HP before a response cue aborted the trial. Two stimuli were presented before the response cue for the first trial and any aborted trials.

A juice reward was delivered during the intertrial interval after approximately 50% of trials with correct responses. The variable reward scheme was selected for two reasons: (1) to maintain engagement in the task, particularly during more difficult blocks of stimuli, and (2) to avoid assumptions as to the discriminability of ICMS stimuli.

After initial training, blocks of auditory stimuli (~1-s duration clips of animal calls, etc.) were added to the task, followed by blocks of single or multichannel ICMS. Four to eight trials of the same stimulus type were grouped together into a block. Within each block, approximately half of the trials presented a “Match” stimulus (same as the previous trial) and half presented a “Change” stimulus (different than the previous trial). Once all trials within a block were attempted, the task continued with a block of a different stimulus type.

### Single-Channel ICMS

During each experimental session where single-channel ICMS was performed, two or three microelectrodes were driven into the somatosensory cortical areas contained within the chamber. The multi-electrode microelectrode drive (NaN Instruments) that we used for these experiments includes a grid which provides for controlled spacing between each of up to 16 separately driven microelectrodes. We always used the spacer, insuring that horizontal spacing between electrodes was always in units of 1 mm in the *X* and *Y* directions. The spacing between electrodes and their location within the chamber varied each day. Electrodes were spaced at least 1 mm, and up to 8 mm, apart; on average, they were separated by 5 mm. This arrangement typically placed one electrode in a region of somatosensory cortex corresponding to the index finger, another in the thumb, and the last on the face.

A single-channel ICMS stimulus consisted of a 1-s duration train of 220-Hz stimulation on a single microelectrode. Each pulse was a cathodal leading, biphasic, symmetric square wave with an amplitude of 65 μA and a duration of 0.2 ms/phase. Stimulation was delivered by a current controlled stimulator running custom software (MS16, Tucker-Davis Technologies). “Match” trials consisted of repeated stimulation of the same electrode on consecutive trials. The electrode to which ICMS was applied shifted between “Change” trials. No stimulation was applied during Null trials.

### Stimulus Encoding for Multichannel ICMS

**Figure 2:**
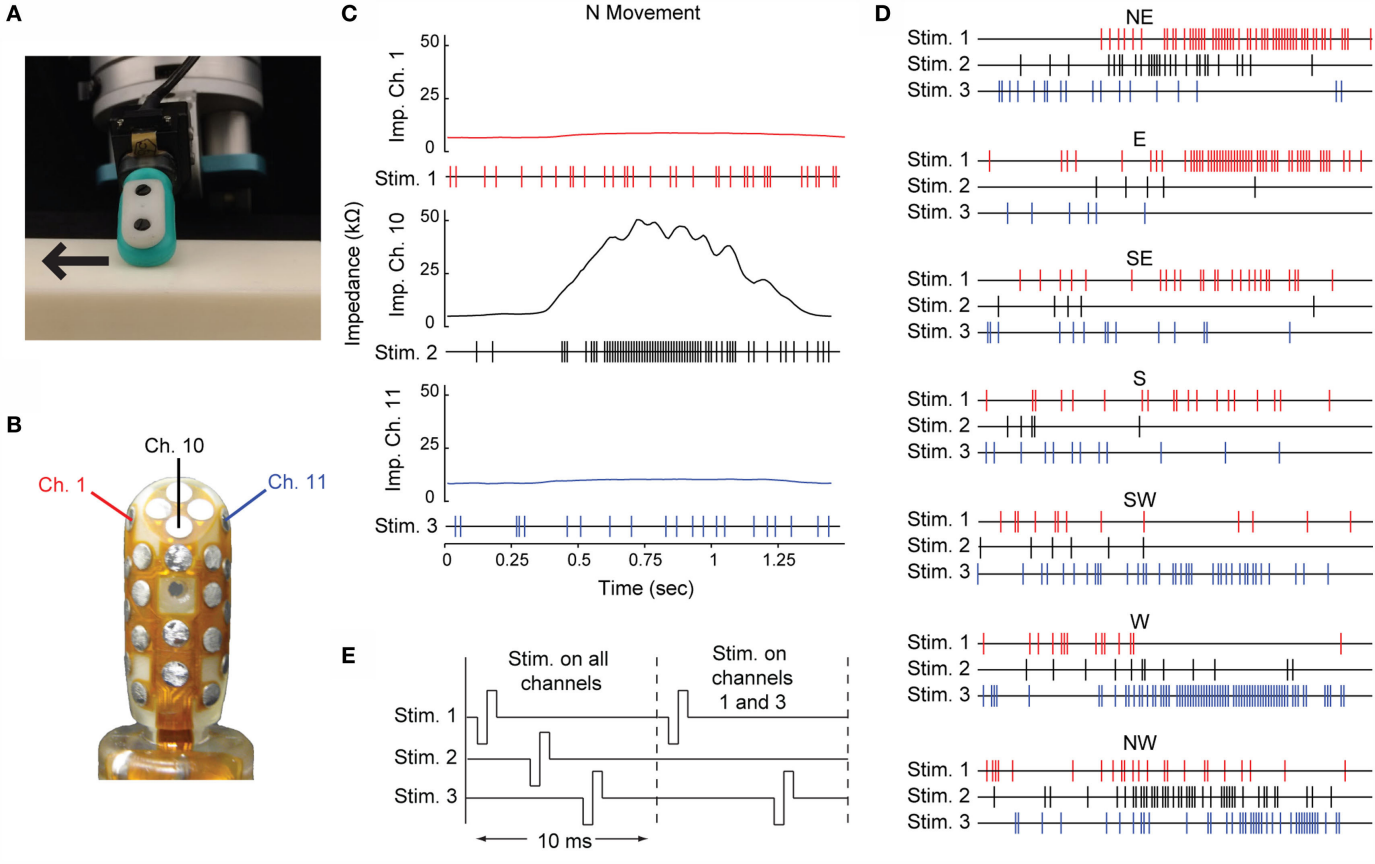
**Multichannel ICMS driven by the BioTac sensor**. **(A)** The BioTac sensor moving across the surface in the East movement direction. **(B)** Three impedance electrodes within the BioTac sensor were chosen to drive stimulation. **(C)** Impedance traces from the three selected channels are shown for the N movement direction. Each of the raw impedance signals were normalized, then the deviation of the signal above or below its baseline was used to assign an instantaneous probability of stimulation at a sampling rate of 100 Hz. In the three resulting stimulation trains for the N movement direction, each hash mark represents a single cathodal-leading, biphasic, symmetric square wave pulse with a duration of 0.2 ms/phase. **(D)** The multichannel stimulation trains for the seven remaining movement directions. **(E)** The stimulation pulses for each channel within a 10-ms time bin were spaced such that no two electrodes passed current at the same time. The timing of each channel’s pulse within the bin remained constant regardless of whether a stimulation pulse was delivered on one, two, or three electrodes in that time bin.

The stimulation trains used for multichannel ICMS in this study were generated by moving a multimodal BioTac sensor (Fishel et al., 2013) across a lightly textured planar surface at a speed of 5 cm/s (Figure 2A). The sensor was mounted on a Barrett WAM robot arm (Barrett Technology, LLC), which brought the sensor to a horizontal planar surface and moved it in one of eight directions, separated by 45° (represented by compass directions – N, NE, E, SE, S, SW, W, and NW). The deformation of the skin of the BioTac’s deformable fingerpad was encoded by the array of impedance sensing electrodes embedded in the rigid core of the sensor.

A threshold was set on the low frequency vibration data collected by the BioTac’s hydrophone to identify the beginning and end of the sensor’s contact with the surface. We selected three impedance channels that displayed the strongest differential responses to movement direction (Figure 2B). For each channel, we subtracted the baseline impedance and normalized the amplitude of the signal relative to its peak response during any movement direction.

We used these normalized impedance signals to construct three probabilistic stimulation trains with a maximum stimulation frequency of 100 Hz, or one pulse every 10 ms. For every 10-ms time bin, the deviation of the normalized impedance value from baseline was determined. A 20% probability of stimulation was assigned to baseline. Decreases in impedance from baseline (resulting from bulging of the skin away from the rigid core of the sensor) reduced the probability of stimulation down to a floor of 0%, while increases in impedance (resulting from compression of the skin toward the core) raised the probability of stimulation within a time bin up to 100%:

$$P{\left(\text{stim}\right)}_{i}=0.2+\left(\frac{x_{i}-x_{b}}{x_{\text{max}}-x_{b}}\right).$$

Here, *P*(stim)*_i_* is the probability of a stimulus in time bin *i, x_i_* is the value of the impedance at time *i, x_b_* is the background impedance (i.e., the impedance when the sensor is not in contact with a surface), and *x*_max_ is the maximum impedance value.

An example of the raw signals from the three selected impedance channels and the resulting stimulation trains are shown in Figure 2C. Stimulation trains corresponding to the other seven movement directions are shown in Figure 2D.

For each movement direction, the three stimulation trains were offset by 2.1 ms and interleaved. This ensured that current was passed through only one electrode at a time and that the temporal information content of each stimulation train was preserved. All stimulation pulses were cathodal-leading, biphasic, symmetric, square waves with an amplitude of 65 μA and duration of 0.2 ms/phase. Examples of the distribution of pulses within each time bin are shown in Figure 2E.

A multichannel ICMS stimulus consisted of a 1-s duration train of stimulation delivered synchronously across three microelectrodes, corresponding to movement of the sensor in one of the eight directions. For “Match” trials, stimulation trains for the same direction of movement were delivered on consecutive trials. A stimulation train corresponding to a different direction of sensor movement, compared to the previous stimulus, was delivered for “Change” trials. Initially, we selected only the most distinct multichannel stimulation trains, corresponding to the four cardinal directions (N, E, S, and W). During later experimental sessions, stimulation trains corresponding to all eight movement directions were presented.

## Results

### General Analysis Methods

**Figure 3:**
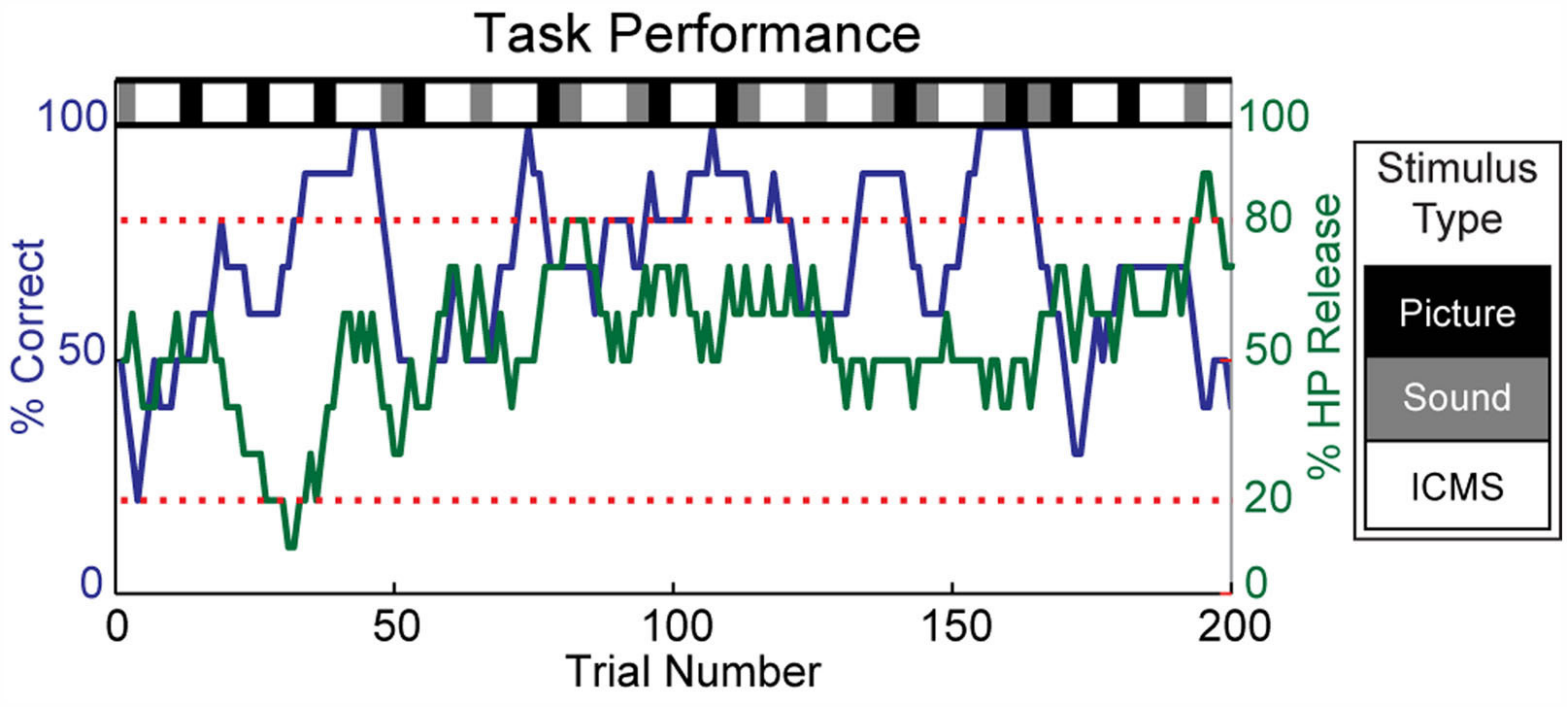
**Overview of task performance**. Within each experimental session, randomized blocks of visual, auditory, and ICMS stimuli were selected, as indicated by the grayscale bar at the top of the plot. The subject’s discrimination accuracy is represented by a 10-trial moving average of correct responses (blue). Streaks of strong performance were often observed, but the overall accuracy varied greatly within an experimental session. The subject’s engagement in the task was monitored by a 10-trial moving average of the number of trials in which the holdpad was released during any phase of the trial or in the intertrial interval (green). Experimental sessions were excluded from analysis when the moving average of holdpad releases remained below 20% or exceeded 80% for more than one-third of the total number of trials. Sessions where fewer than five blocks of ICMS were completed were also excluded from analysis.

The animal subject learned to perform the discrimination task successfully and typically completed several hundred trials during each experimental session with few aborted trials. Figure 3 shows the percentage of correct responses for a 10-trial moving average throughout one experimental session. Blocks of visual and auditory stimuli were usually completed with few errors; there were also periods of strong performance on ICMS trials, but they were often intermixed with strings of incorrect responses.

The monkey clearly found the discrimination of ICMS stimuli to be a difficult task. At times, the subject significantly altered his frequency of response due to disinterest or agitation. Based on the animal’s behavior, we used a 10-trial moving average of the rate of HP release (during any phase of the trial or during the intertrial interval) as a measure of the subject’s engagement with the task. Since 50% of the trials presented Match conditions that did not require HP release, a continuous deviation from a 50%-HP release rate indicated a change in the animal’s strategy that was unlikely to correspond to true discrimination. We excluded experimental sessions from our analysis when the HP release rate remained below 20% or above 80% for more than one-third of the total number of trials. Additionally, we excluded experimental sessions where the subject completed fewer than five blocks of ICMS (<20–40 trials). We analyzed the discrimination of discrete stimulation on single electrodes in 31 experimental sessions and multichannel stimulation across several electrodes during 26 experimental sessions.

For the remainder of the analysis, we focused on the discriminability of ICMS stimuli, thus trials with visual and auditory stimuli were removed. The probability of the monkey randomly selecting the correct response for each trial was 50% because approximately equal numbers of Match and Change trials were presented over the course of the experiment. Due to this task design, reporting a raw percentage of correct responses does not provide sufficient information to judge whether the pattern of responses is significantly different from chance levels of performance. In our first approach to the data, we carried out an ROC analysis and the results for ICMS were consistently above the unity line, indicating that the true positive rate was consistently higher than the false positive rate. However, that difference was very small, leading us to analyze the data in terms of cumulative probability densities rather than *d*′.

The cumulative probability densities here report the probability of observing up to *X* correct responses during *N* trials when the probability of success on each trial is 50%. A value of 0.95 or greater indicates that true probability of success was significantly greater than 50% (*p* = 0.05), indicating that the animal was using ICMS to cue its responses. The use of the cumulative probability density also allows more direct comparisons between experimental sessions as it minimizes the effects of unequal trial numbers.

### Single-Channel ICMS Discrimination

The subject learned to discriminate single-channel ICMS stimuli in only a few experimental sessions. However, there was substantial variability in discrimination performance between experimental sessions, with some low discrimination performance occurring well after the animal was trained. This variability may be due to the daily change in the position and depth of the electrodes – the subject had to learn to identify and discriminate new stimuli each day. We did not tune the stimulation amplitude for each electrode; differences in detectability or perceived strength of stimulation on one or more of the electrodes could also have affected discrimination accuracy in this manner.

**Figure 4:**
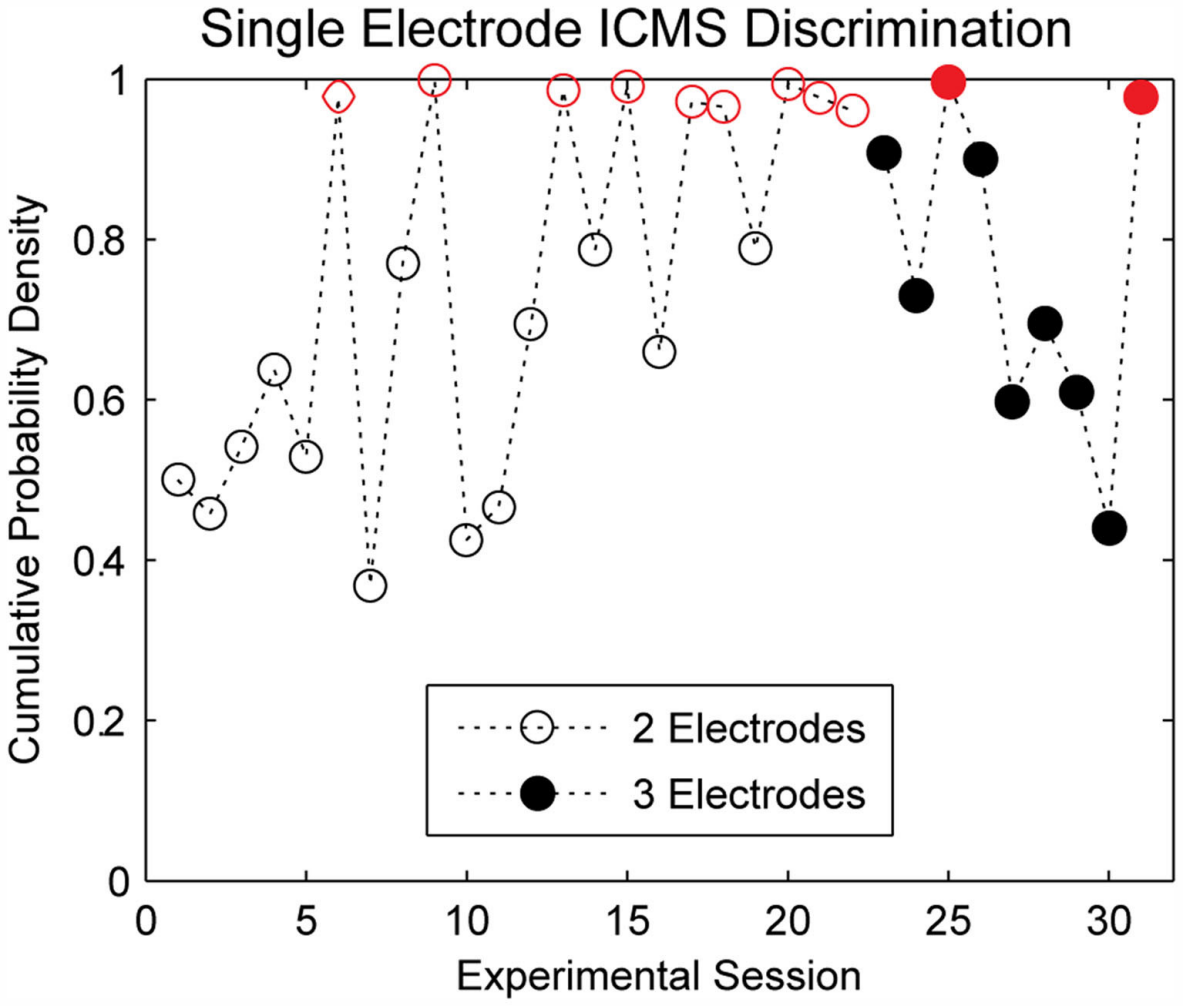
**Discrimination accuracy for single electrode ICMS**. The subject quickly learned to perform the discrimination task; however, there was considerable variability in performance across experimental sessions. Performance on stimulation trials was significantly better than chance levels (*p* < 0.05, indicated by open or closed circles in red) for 7 of 22 sessions where stimulation was delivered across two electrodes and two of nine sessions where stimulation was delivered across three electrodes.

The cumulative probability densities for experimental sessions where single-channel ICMS was delivered are shown in Figure 4. In each trial, a train of 220-Hz stimulation was applied to one of two electrodes during the first 22 experimental sessions; seven of these experimental sessions showed performance significantly better than chance (*p* < 0.05). For the remainder of the single-channel ICMS sessions, the same stimulation train was applied to one of three electrodes during each trial. Performance on two of these nine sessions exceeded the threshold for statistical significance (*p* < 0.05).

**Figure 5:**
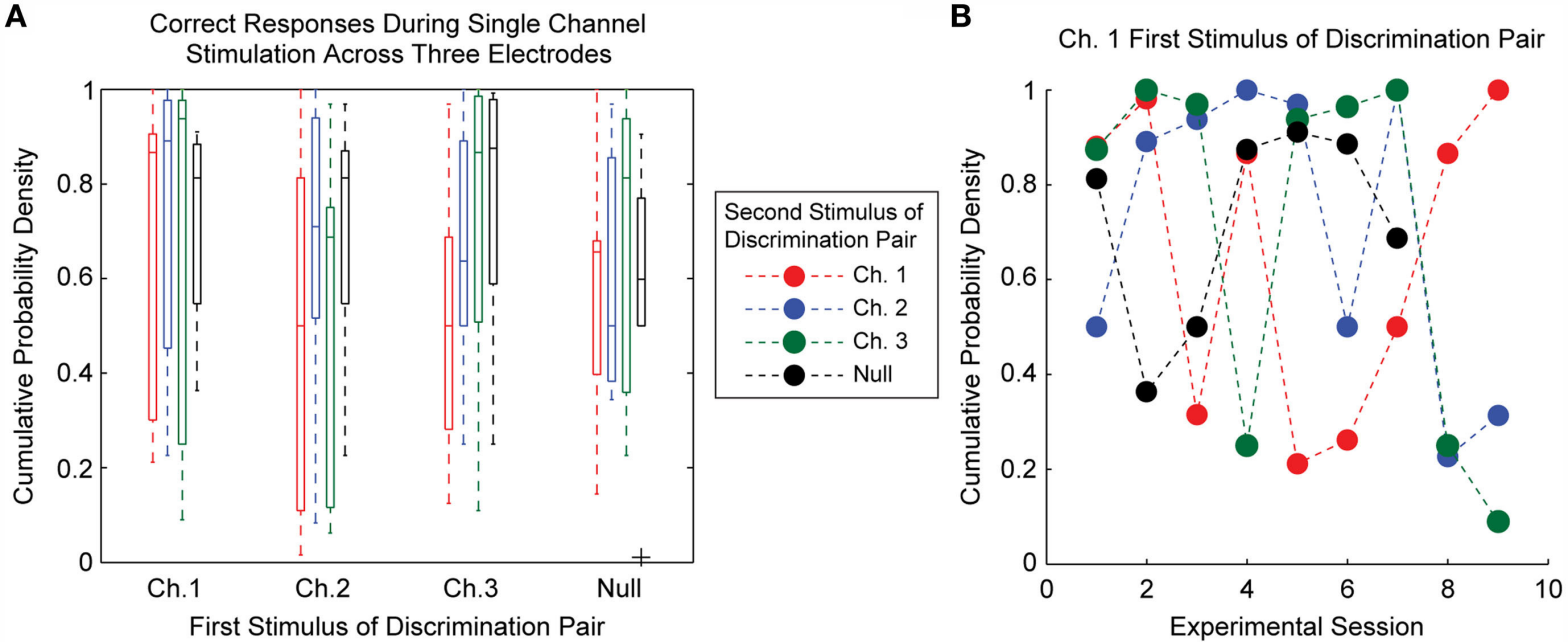
**Detail of single electrode discrimination performance for separate stimulation on three electrodes**. **(A)** The boxplots represent the accuracy of discrimination for each possible combination of stimulus pairs over all experimental sessions. Each stimulus pair had high variability in the discriminability between experimental sessions. Stimulation order appears to have an effect on the discriminability of certain stimuli. **(B)** During a single experimental session, not all stimulus pairs were equally discriminable. This figure demonstrates that the most discriminable pairs of stimuli shifted from day to day. Highly accurate discrimination of all stimulus pairs was not achieved during any experimental session.

The probability of the animal performing a correct discrimination on a trial varied according to the stimuli being compared. Figure 5A shows the cumulative probability densities for each possible combination of single-channel stimuli across all three single-channel ICMS experimental sessions. One key finding here is that the order of stimulus presentation affected discrimination accuracy. For example, the median cumulative probability density for trials comparing stimulation first delivered on Channel 1 and second on Channel 3 was 0.94; this value was 0.5 when the stimuli were reversed.

The pairs of stimuli that were most accurately discriminated differed between experimental sessions, as demonstrated in Figure 5B. Typically, for each electrode and for each experimental session, at least one combination of stimulus pairs was poorly discriminated. We found no systematic differences in the location of the electrodes within the somatotopic map or the depth of electrodes within cortex that could account for this pattern. This finding suggests that ICMS on neighboring electrodes can affect the sensory percepts evoked by ICMS, making discrimination between ICMS delivered at multiple locations a fundamentally difficult task.

### Multichannel ICMS Discrimination

**Figure 6:**
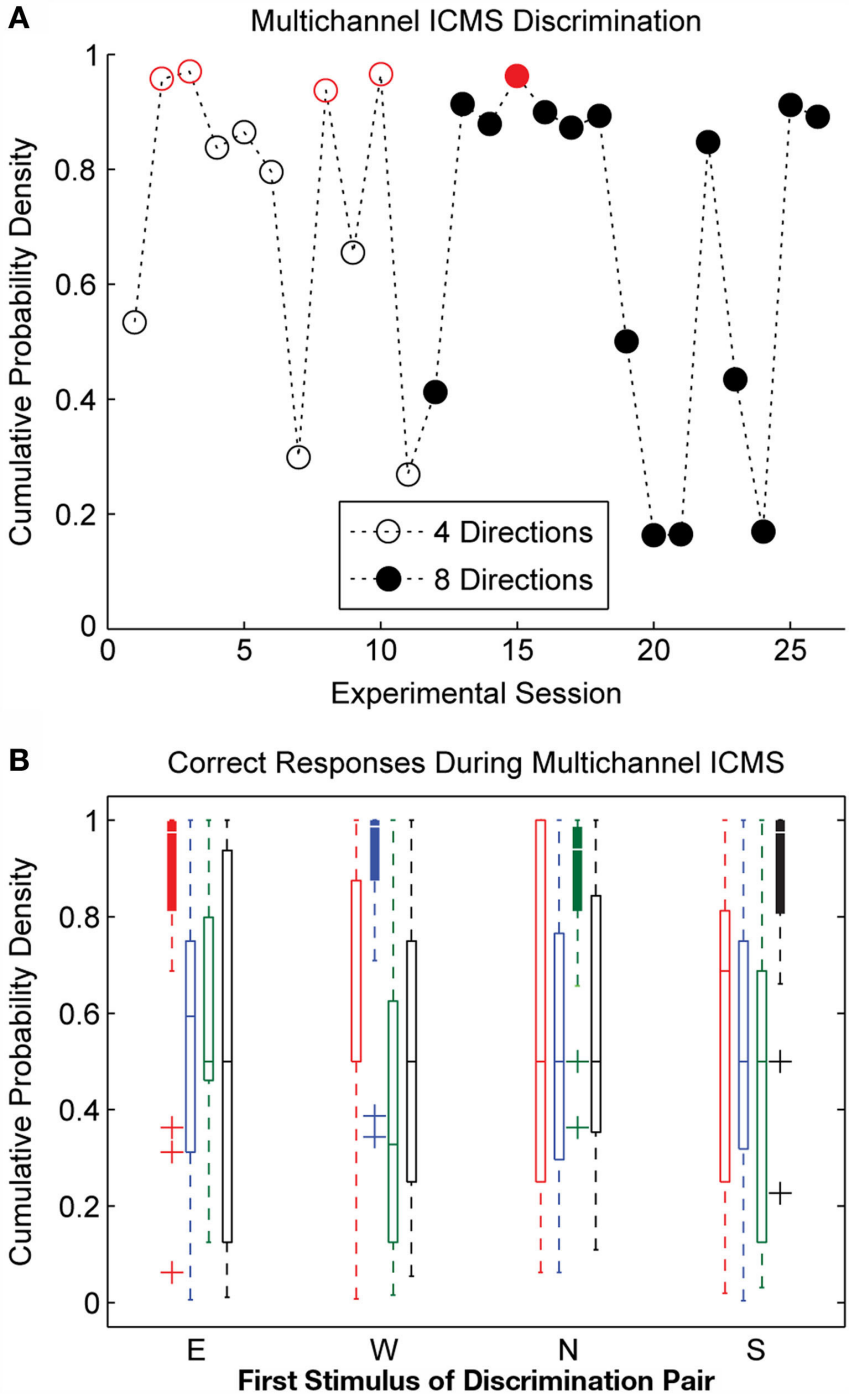
**Discrimination accuracy for multichannel ICMS delivered across three electrodes**. **(A)** Considerable variability in the day-to-day discriminability of multichannel ICMS was observed. Performance on stimulation trials was significantly better than chance levels (*p* < 0.05, indicated by open or closed circles in red) for 5 of 26 experimental sessions. **(B)** Overall discrimination accuracy during multichannel ICMS trials was lower than for single-channel stimulation. Accuracy at detecting Match conditions (repeated pattern of stimulation on consecutive trials, represented by filled box plots) was significantly higher than the correct detection of Change trials during multichannel ICMS.

Many of the observations for single-channel stimulation also apply to the multichannel ICMS experimental sessions. The overall discrimination accuracy for multichannel stimulation, shown in Figure 6A, was similar to that observed during single-channel stimulation. The initial 11 multichannel experimental sessions utilized only 4 of the 8 sensor movement directions; 3 of these sessions showed discrimination performance significantly better than chance (*p* < 0.05). The remaining 15 sessions drew stimulation patterns from all 8 movement directions. Only one of these experimental sessions met the threshold for statistical significance at a *p*-value of 0.05, and discrimination performance was better than chance at a lower significance threshold (*p* = 0.1) for three additional experimental sessions.

Discrimination performance was greatest for the multichannel stimulation trains for the four cardinal directions (N, E, S, and W). During N, E, and W stimulation trains, the majority of the stimulation pulses were delivered on a single electrode; thus, these stimuli were similar to the single-channel ICMS delivered in earlier experimental sessions. Under the carefully controlled conditions utilized by the robot in this experiment, the BioTac sensor did not strongly encode movement in the S, SE, and SW directions; thus, few stimulation pulses were delivered during these stimuli, approximating the Null condition from single-channel ICMS sessions. The majority of the pulses of stimulation trains corresponding to the NE and NW movement directions were distributed across two electrodes. This pattern of stimulation appeared to be more difficult for the animal to discriminate.

Similar to the single-channel ICMS experimental sessions, discrimination performance on various combinations of stimuli varied greatly from day to day. On average, the discrimination accuracy for multichannel ICMS sessions was lower than for single-channel ICMS. However, as shown in Figure 6B, Match trials were correctly discriminated with high accuracy during multichannel ICMS sessions. The discrimination accuracy for Match trials was significantly higher than for Change trials (*p* ≪ 0.05). The correct identification of Match trials cannot be attributed to a global change in the animal’s response strategy, as experimental sessions were excluded if the HP release rate dropped below 20% during a substantial portion of the experiment.

## Discussion

In this series of experiments, we delivered single and multichannel ICMS *via* electrodes spaced within somatosensory cortex and examined the ability of a non-human primate to discriminate the resulting sensations. Our results indicate that stimulation on single electrodes spaced by at least 1 mm within somatosensory cortex can produce sensations that are discriminable. Some trains of multichannel stimuli may also produce discriminable sensations; in this experiment, the subject most readily identified matching multichannel ICMS stimuli. This study suggests that delivering multiple independent and discriminable streams of sensory information *via* ICMS is a very challenging task.

This work is the first attempt to use ICMS of somatosensory cortex to simultaneously communicate multiple independent streams of information directly from a tactile sensor. This capability will be necessary for intuitive closed-loop control of a neuroprosthetic limb. It remains unclear if ICMS is capable of producing sensations of the necessary quality, robustness, and resolution for this application. Here, we discuss some of the factors that may have affected our experiments and suggest directions for future related work.

### Factors Affecting the Discriminability of ICMS Stimuli

The most notable difference between this experiment and other studies of ICMS discriminability is our use of acute electrodes instead of a chronically implanted electrode array (Schmidt et al., 1996; Otto et al., 2005; O’Doherty et al., 2012; Berg et al., 2013; Kim et al., 2015). We chose to use acute electrodes because they allowed us to separate stimulation sites over a larger distance, presumably increasing the probability of evoking multiple distinct sensations *via* ICMS. However, since the position of the electrodes and their depth within cortex varied daily, the ICMS-induced sensations are also expected to have varied significantly. Other studies have demonstrated that animals learn to better detect and discriminate ICMS stimuli over a period of days to weeks if a fixed electrode array is utilized (O’Doherty et al., 2011, 2012).

Poor detectability of ICMS may have also affected the subject’s performance on stimulation trials. During some experimental sessions, the monkey responded to stimulation with great conviction that suggested a robust sensation was produced; performance was typically greatest during these experimental sessions. Some highly accurate discrimination was performed on days when the monkey’s responses to stimulation were more tentative, but it is possible that these sensations were more difficult for the subject to detect. We did not attempt to identify a stimulation threshold for each electrode during each experimental session as it would have added considerable complexity to what was evidently already a difficult task for the animals. Instead, we used a fixed stimulus strength and operated on the assumption that we were generally well above threshold. However, it is probable that variations in the detectability of stimulation on different electrodes could account for some of the difficulties we observed. The fixed stimulation amplitude we utilized is lower than some reported ICMS thresholds in somatosensory cortex (Berg et al., 2013); however, the subject’s response to ICMS at these levels suggests that the amplitude was generally sufficient to evoke a sensory percept.

Although ICMS initiates neural activity within milliseconds, stimulation experiments in humans have suggested that conscious perception of the sensation elicited by stimulation is much slower (Libet, 1973). Our task required the subject to identify changes in the features of the stimulation-induced sensation. The rigidly timed structure of this task may not have allowed sufficient time for the animal to make these judgments before a secondary stimulus was delivered or a response was required. For this reason, more flexible tasks that permit active haptic exploration may be preferable for experiments that require discrimination of multiple ICMS stimuli (O’Doherty et al., 2011; Thomson et al., 2013).

In much of the prior work on the use of ICMS to deliver tactile sensation, electrode arrays were chronically placed providing that stimuli are reliably delivered to the same location over the span of weeks [e.g., see O’Doherty et al. (2011), Tabot et al. (2012), and Kim et al. (2015)]. In these cases, an animal has time to learn how to interpret a fixed stimulation, and the neural systems can adapt to that stimulation to provide a more robust response to that ICMS. However, even when an electrode remains in a fixed position within cortex and identical ICMS stimuli are delivered, the sensations elicited by stimulation may change over time. Repeated stimulation on a single electrode can affect the minimum amplitude required to evoke a sensation due to short term facilitation (Libet, 1973; Schmidt et al., 1996; Tehovnik et al., 2005). By similar neuroplastic processes, recent stimulation on other nearby electrodes can also modify the size, location, or quality of sensations evoked by ICMS (Libet et al., 1964; Bak et al., 1990; Schmidt et al., 1996). These effects may have reduced the detectability or discriminability of ICMS stimuli during this experiment. Although we made efforts to maximize the time between ICMS trials, our results may have been influenced by these temporal effects.

### Implications for Bidirectional Neuroprostheses

The development of a closed-loop, sensory capable neuroprosthesis requires innovation in the design of the limb, the encoding of relevant tactile and proprioceptive information, and methods for delivering that information to the user. To provide a functional benefit to the user, such a device would need to relay real-time sensory information corresponding to at least two opposing digits.

Most commercially available prostheses have not yet integrated sensors capable of encoding touch and posture into the body of the device. Constraints on the size, weight, and computational power will affect the choice of components on a sensorized prosthesis. The BioTac and other similar sensors are well-suited to provide tactile information for this application because they combine several sensor modalities into a compact package.

The impedance signals from the BioTac were translated into multichannel ICMS with high fidelity by the methods we utilized. We expect this encoding scheme to scale easily to expand the number of stimulation electrodes, and this method can be used to generate stimulation trains in real time. In this study, information that was clearly encoded by the impedance sensor was represented by modulation of the stimulation frequency on the corresponding electrode. In natural sensation, intensity is primarily encoded by the frequency of action potentials; thus, our multichannel ICMS trains are expected to convey similar characteristics of the signal, although some high frequency information content is sacrificed by this method. Other research groups have chosen to encode intensity by modulating the amplitude of stimulation (Berg et al., 2013), which may produce a more robust signal by activating a larger group of neurons. A combination of these encoding schemes may provide the most effective source to drive sensory ICMS.

We have demonstrated in this work that the sensations elicited by single or multichannel ICMS of somatosensory cortex can be difficult to detect or discriminate. Similar challenges have been reported in other sensory cortical areas as well (Schmidt et al., 1996). Although our current understanding of ICMS is not sufficient to enable delivery of robust and high-resolution sensory feedback at this time, stimulation can clearly be used to communicate more than one stream of simple tactile information in real time. The fundamental limit on the resolution of stimulation-induced sensation appears to be not in the spacing of electrodes within cortex but in the simultaneous generation of multiple, stable, and focal sensations.

The characteristics of sensations evoked by natural stimuli and ICMS can differ greatly; this can largely be explained by differences in the pattern of activation of somatosensory cortical neurons. Ascending fibers transmitting sensory information from the periphery form synapses on neurons in cortical layer IV (Schwark and Jones, 1989). Activation of these neurons initiates a cascade of neural activity that is primarily restricted to a vertical column of cortical tissue (Mountcastle, 2005). Conversely, ICMS activates neurons whose axons pass near the active electrode site. This non-specific recruitment is expected to simultaneously activate both inhibitory and excitatory neurons in multiple cortical layers and across multiple cortical columns (Brock et al., 2013; Overstreet et al., 2013). Because of these differences in the pattern of neuronal recruitment, naturally evoked sensations are typically more focal and consistent than ICMS-induced sensations.

Despite these difficulties, ICMS can be carefully designed to provide sensation that has distinguishable temporal and spatial characteristics. In one study, animals reported that ICMS at different locations on a chronic array elicited sensations on either the left or right side of their palm (Tabot et al., 2012). This provides some evidence that ICMS within somatosensory cortex can produce sensations with reasonable spatial selectivity, although a correction to that study indicates a caveat about using ICMS, as one of their two subjects had substantial difficulty with the task. This difficulty may have been due to a failed device for providing ICMS, although this is not entirely clear from the results presented by the authors.

By contrast, using a peripheral approach has many advantages. In addition to being a significantly less invasive surgical procedure, peripheral nerve stimulation also takes advantage of the neural circuitry, which already exists between peripheral afferents, and the cerebral cortex. There is substantial convergence and divergence between receptor systems and receptor types as the signals ascend from the periphery. This recombination of signals provides a basis for extracting sensory representations that are not contained in any single afferent type [see Johansson and Flanagan (2009) for a review]. Cortex in adults is clearly adapted to accepting those processed signals and using them to interpret the interactions between the body and the world. Given these considerations, we expect that ICMS would be used in bidirectional neuroprostheses only as a last resort.

Over time, neuroplasticity may improve the utility of sensations elicited by ICMS; for example, the sensations may change in strength or quality, become more focal, and correspond to a particular body location more consistently. To capture the benefits of this neural process, stimulation must be applied consistently, spatially, and temporally, in behaviorally relevant situations (e.g., tactile exploration or grasping). Ideally, such stimulation will provide a real-time response that can create a functional sensory feedback loop. ICMS studies in human subjects are likely to provide a wealth of information about the localization, quality, and intensity of sensations evoked by stimulation that is difficult to obtain from animal models due to their abstract nature. Moving forward to such studies would provide extremely valuable insight into the development of maximally effective ICMS techniques.

## Author Contributions

CO, ST, and VS conceived and designed research project. RH, RW, and VS collected tactile sensor data. CO performed experiments. CO and ST analyzed data and interpreted results. CO prepared figures and drafted manuscript. All the authors reviewed, revised, and approved final version of the manuscript.

## Conflict of Interest Statement

The authors declare that the research was conducted in the absence of any commercial or financial relationships that could be construed as a potential conflict of interest.
